# Supplementary material for: Modeling the Kinetics of Polyethylene Terephthalate and Polyesters with Terminal Hydroxyl Groups Transesterification Reactions
Source: Polymers (Basel). 2025 Apr 6;17(7):992. doi: 10.3390/polym17070992 (PMC11991440; doi:10.3390/polym17070992)
Supplement: Supplementary file 1 [file polymers-17-00992-s001.zip › Table S1. HNMR ODEET data..pdf]

Table S1. HNMR ODEET data.

Sample	Time	A(EE)	A(ED)	A(DD)	RD exp	X(E) exp	X(D) exp	RD sim	X(E) sim	X(D) sim	$\left\lvert\frac{RD\ exp - RD\ sim}{RD\ exp}\right\rvert$	$\left\lvert\frac{X(E)\ exp - X(E)\ sim}{X(E)\ exp}\right\rvert$	$\left\lvert\frac{X(D)\ exp - X(D)\ sim}{X(D)\ exp}\right\rvert$
ODEET-75:25	7.5	55.43	29.09	15.48	0.69	20.79	48.44	0.74	22.14	51.99	0.07	0.07	0.07
	15	48.28	41.26	10.46	0.96	29.94	66.36	0.94	28.06	65.91	0.02	0.06	0.01
	30	47.72	44.43	7.85	1.06	31.77	73.89	1.05	31.49	73.98	0.00	0.01	0.00
	60	44.15	46.47	9.38	1.06	34.48	71.24	1.09	32.42	76.19	0.03	0.06	0.07
					Average approximation error			**2.48**	**3.93**	**3.01**	*0.12*	*0.20*	*0.15*
ODEET-50:50	7.5	30.35	33.98	35.67	0.68	35.89	32.26	0.70	38.87	30.93	0.02	0.08	0.04
	15	27.44	39.44	33.11	0.79	41.82	37.33	0.77	42.95	34.18	0.03	0.03	0.08
	30	27.83	39.26	32.92	0.79	41.36	37.35	0.78	43.41	34.55	0.01	0.05	0.08
	60	24.12	40.18	35.70	0.81	45.44	36.01	0.78	43.42	34.55	0.04	0.04	0.04
					Average approximation error			**2.03**	**4.09**	**4.82**	*0.10*	*0.20*	*0.24*
ODEET-25:75	7.5	11.03	28.43	60.54	0.71	53.82	16.89	0.77	60.71	16.46	0.09	0.13	0.03
	15	9.26	31.42	59.32	0.84	62.92	20.94	0.86	68.00	18.42	0.03	0.08	0.12
	30	9.61	30.64	59.75	0.82	61.45	20.41	0.88	69.33	18.77	0.08	0.13	0.08
	60	9.30	30.93	59.77	0.83	62.45	20.56	0.88	69.37	18.78	0.06	0.11	0.09
					Average approximation error			**5.20**	**8.96**	**6.25**	*0.26*	*0.45*	*0.31*
